# Supplementary figures and images for: Long-term temporal evolution of extreme temperature in a warming Earth
Source: PLoS One. 2023 Feb 1;18(2):e0280503. doi: 10.1371/journal.pone.0280503 (PMC9891510; doi:10.1371/journal.pone.0280503)

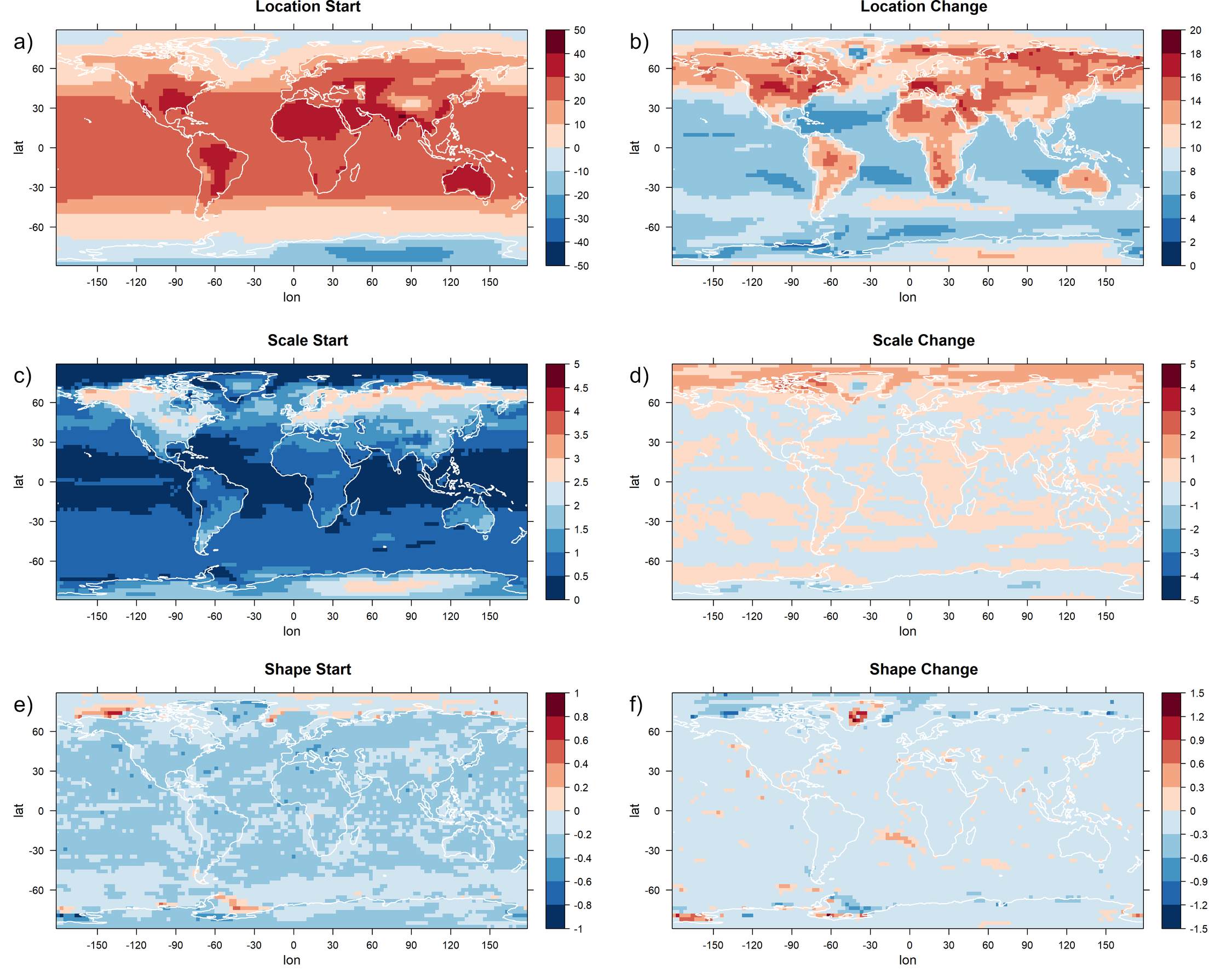

Supplement: S1 Fig — (TIF) [file pone.0280503.s001.tif]

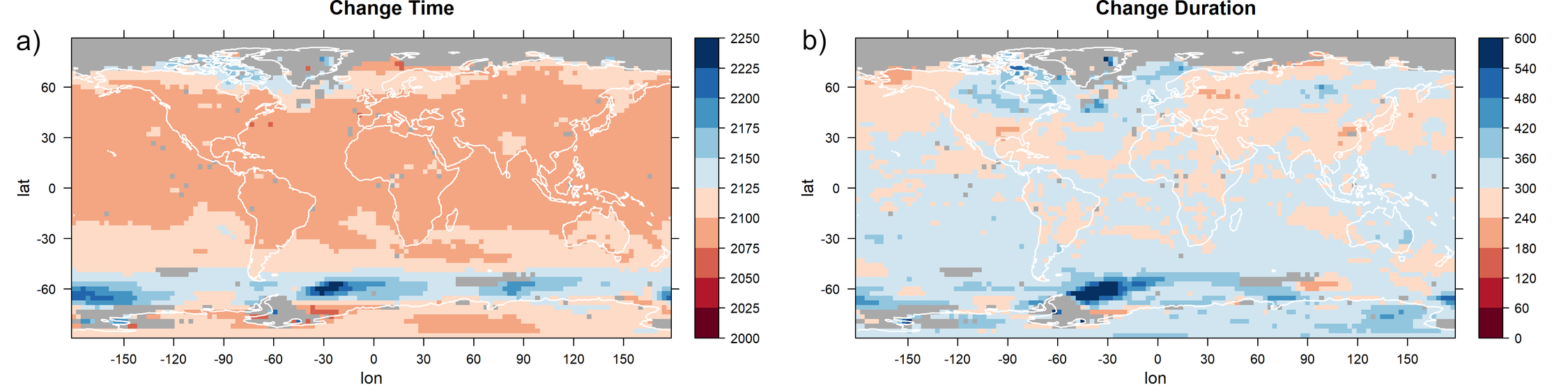

Supplement: S2 Fig — (TIF) [file pone.0280503.s002.tif]

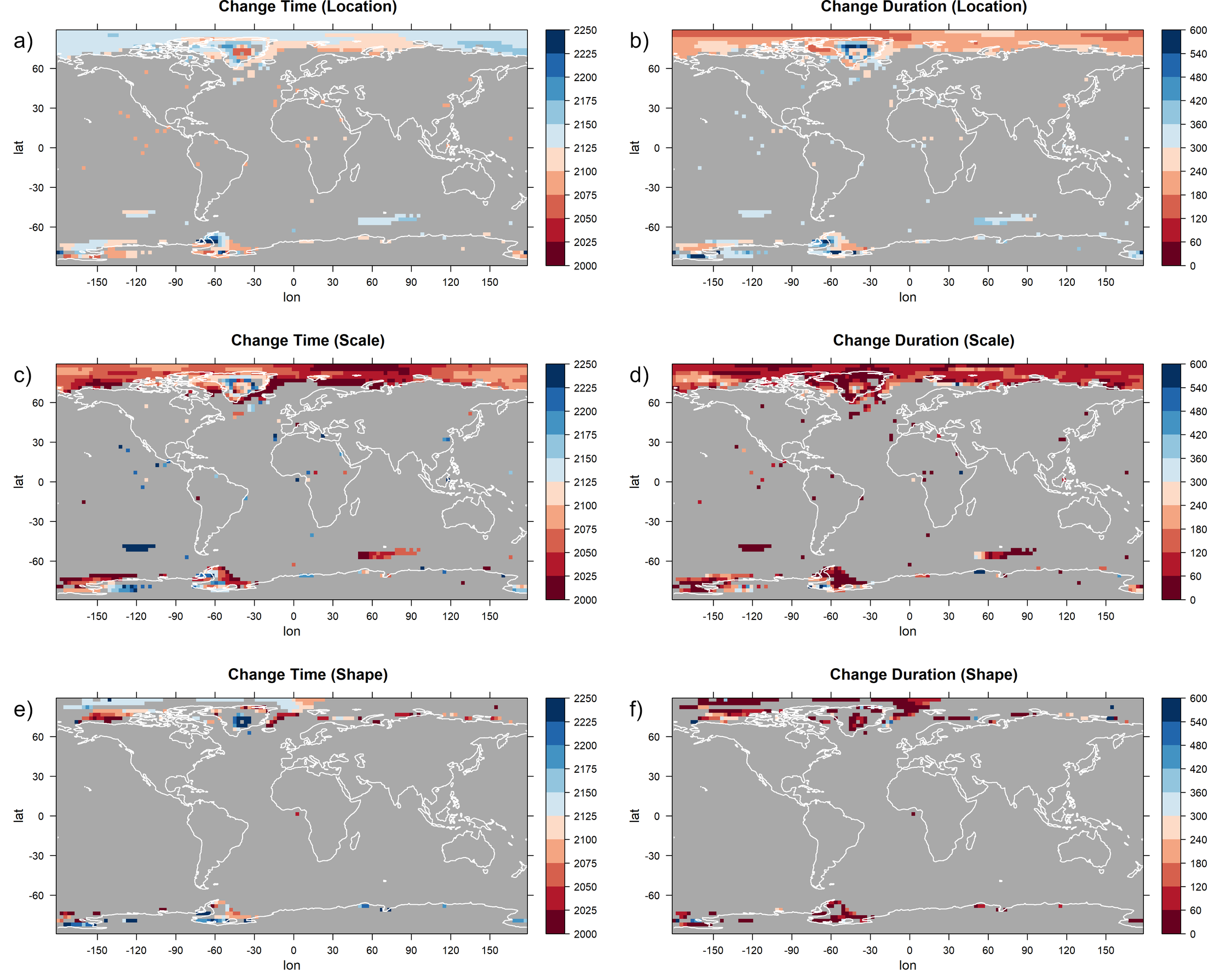

Supplement: S3 Fig — (TIF) [file pone.0280503.s003.tif]

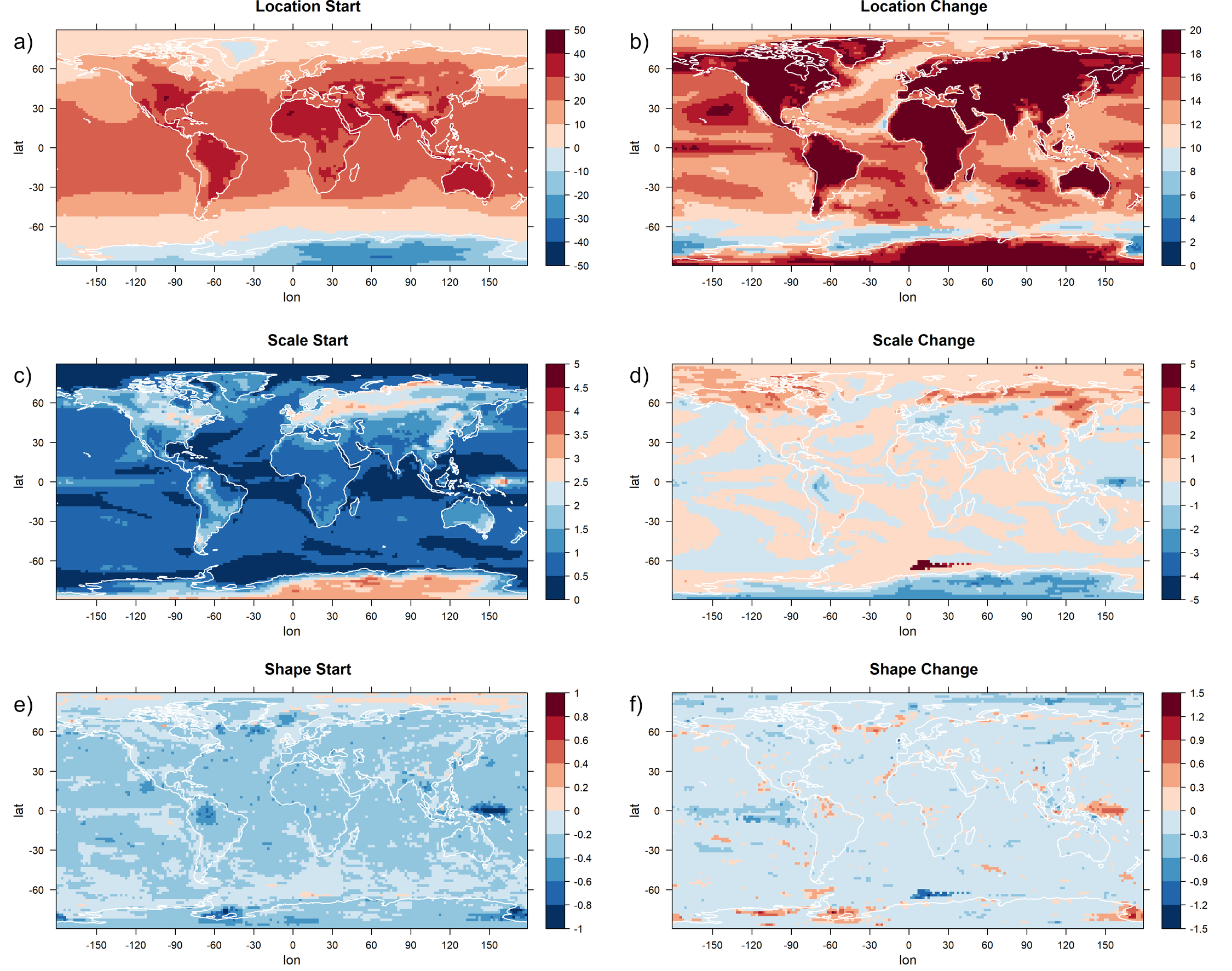

Supplement: S4 Fig — (TIF) [file pone.0280503.s004.tif]

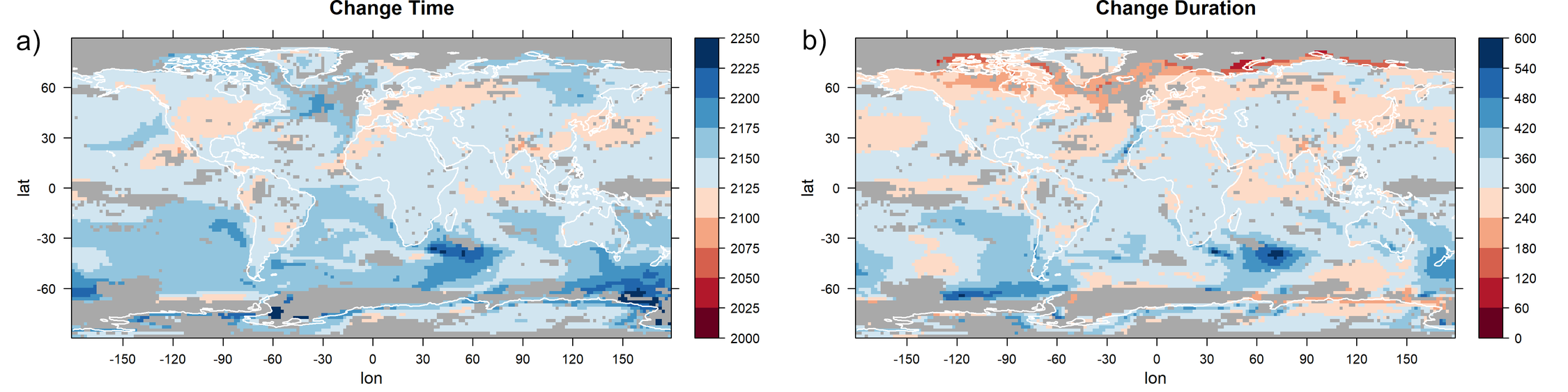

Supplement: S5 Fig — (TIF) [file pone.0280503.s005.tif]

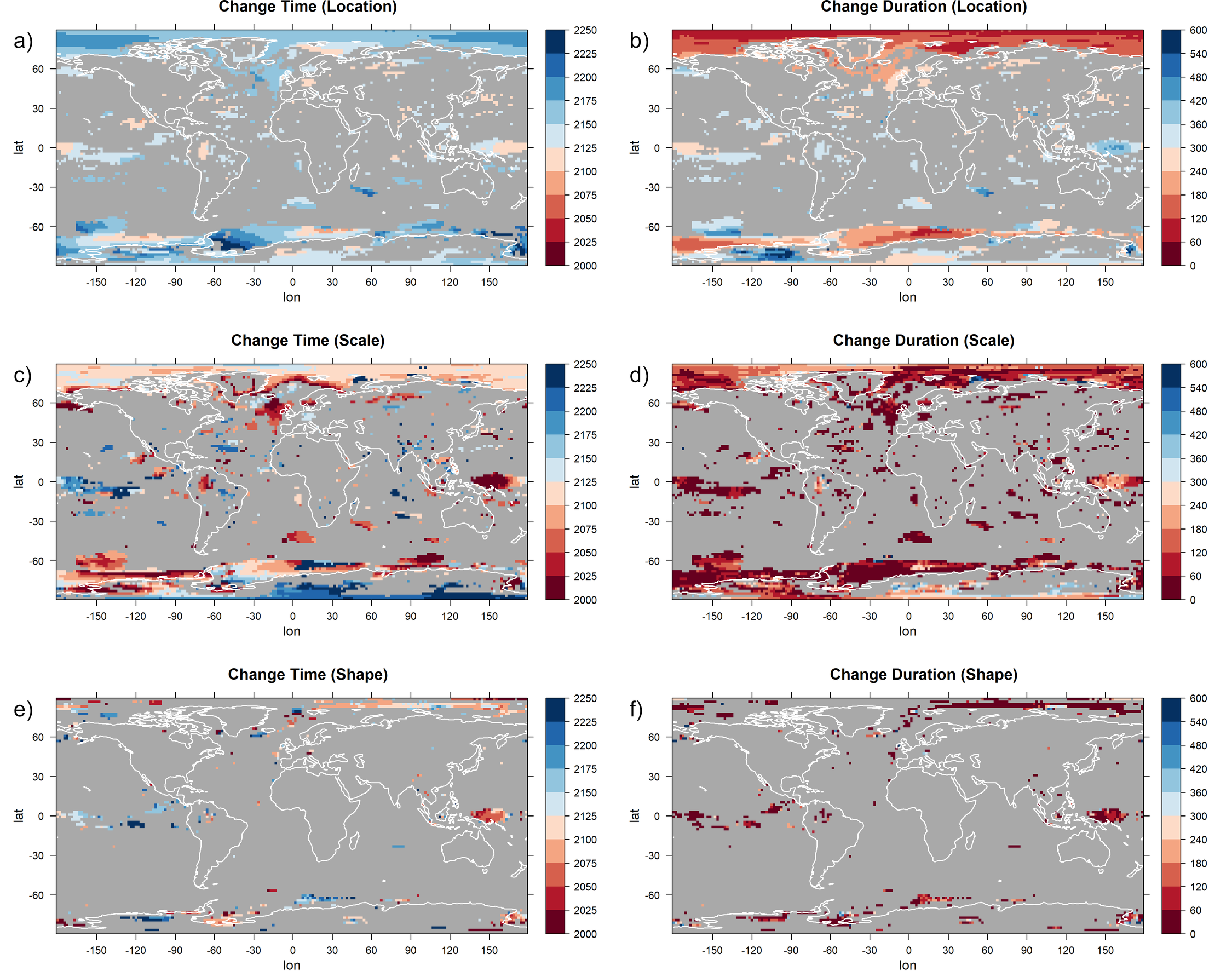

Supplement: S6 Fig — (TIF) [file pone.0280503.s006.tif]

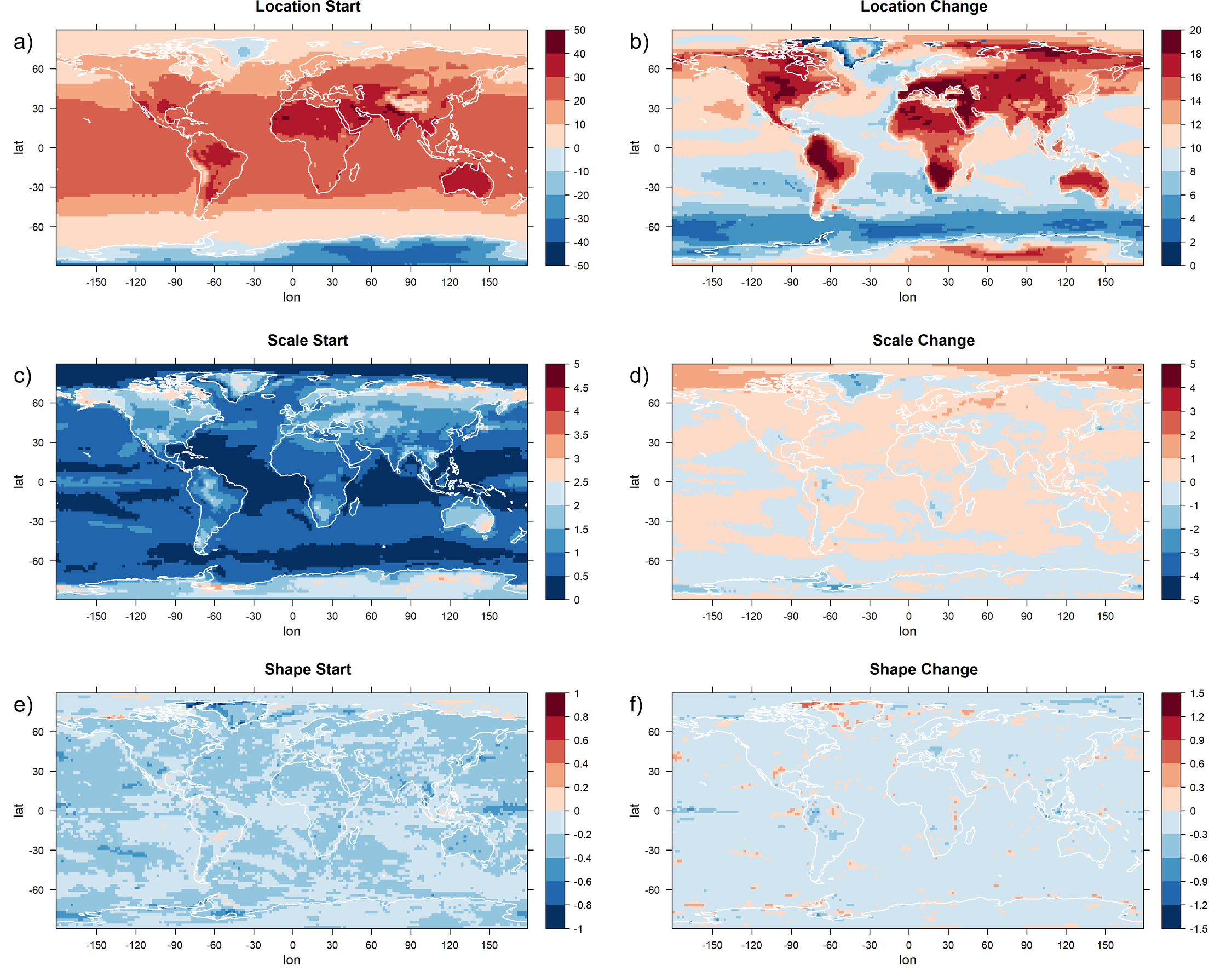

Supplement: S7 Fig — (TIF) [file pone.0280503.s007.tif]

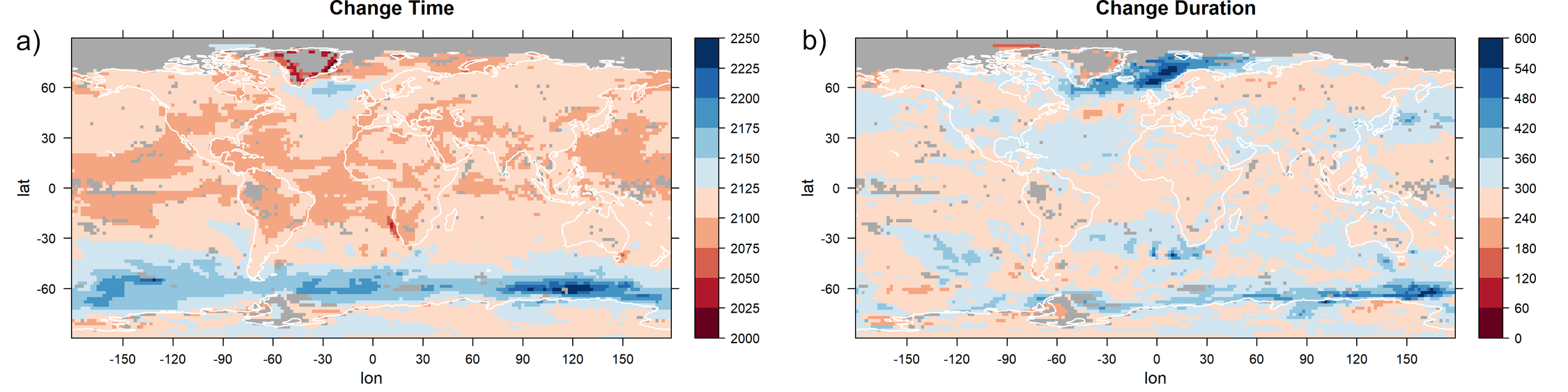

Supplement: S8 Fig — (TIF) [file pone.0280503.s008.tif]

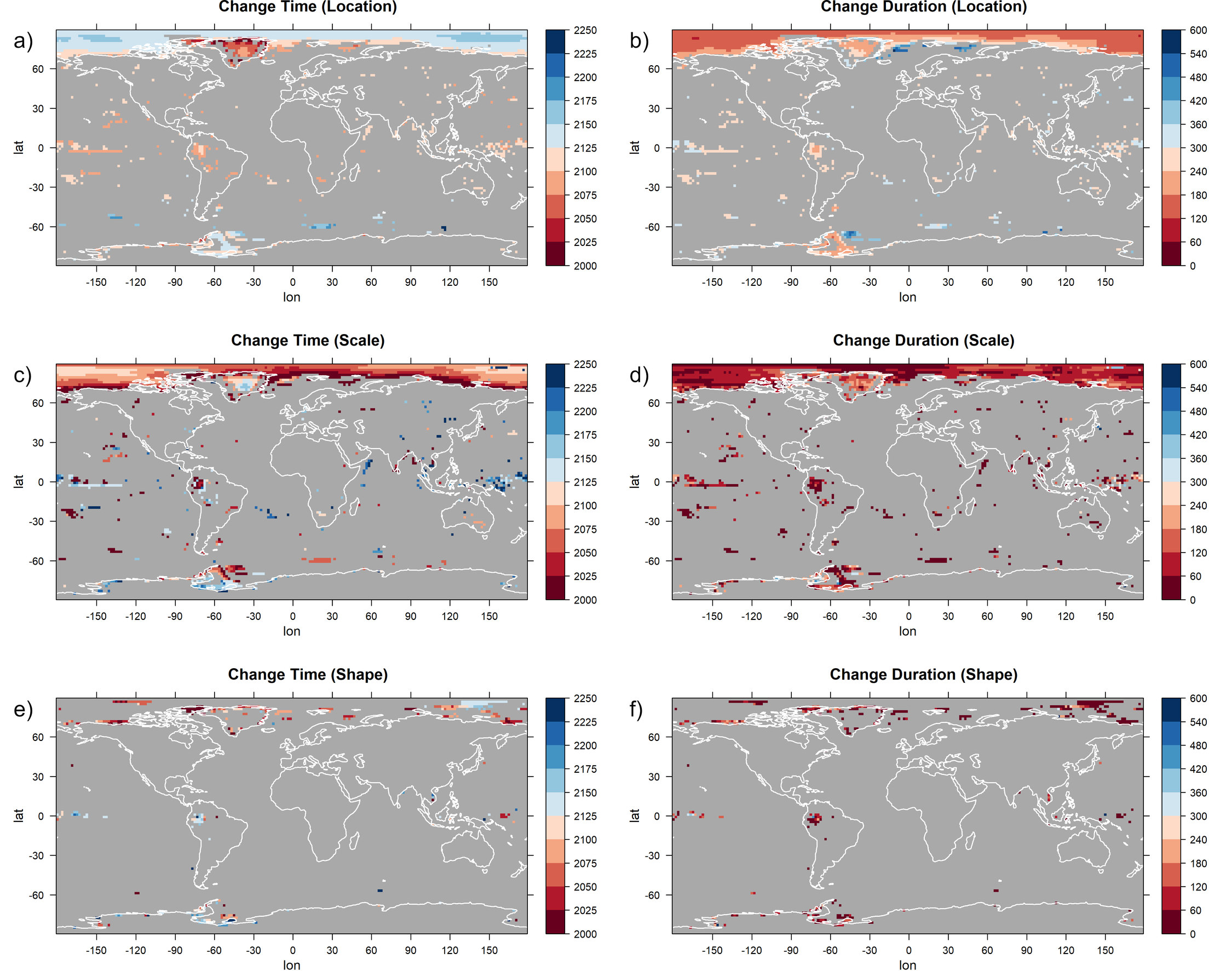

Supplement: S9 Fig — (TIF) [file pone.0280503.s009.tif]
