# Supplementary material for: Long-term temporal evolution of extreme temperature in a warming Earth
Source: PLoS One. 2023 Feb 1;18(2):e0280503. doi: 10.1371/journal.pone.0280503 (PMC9891510; doi:10.1371/journal.pone.0280503)
Supplement: Attachment — Submitted filename: Response to Reviewers.pdf [file pone.0280503.s010.pdf]

Dear editor, dear reviewers,

thank you very much for the revision of our manuscript and the constructive comments. Please find the answers to your remarks below (in blue text color).

Editor Comments

1. Please ensure that your manuscript meets PLOS ONE's style requirements, including those for file naming. [...]

We have adapted the manuscript to the style requirements. The file names of the figure files have been changed according to the guidelines. The author and affiliations section has also been reworked to fit the style guide.

2. Please note that PLOS ONE has specific guidelines on code sharing for submissions in which author-generated code underpins the findings in the manuscript. [...]

We have now uploaded the code to a public repository and included a reference to it in the "Data Availability" statement.

3. We note that you have stated that you will provide repository information for your data at acceptance. Should your manuscript be accepted for publication, we will hold it until you provide the relevant accession numbers or DOIs necessary to access your data. If you wish to make changes to your Data Availability statement, please describe these changes in your cover letter and we will update your Data Availability statement to reflect the information you provide.

As a consequence of the revision, the data used in the manuscript have changed. All data used are now publicly available, and we have referenced them with a DOI in the updated "Data Availability" statement of the revision. We have also described this change in the cover letter.

4. We note that Figures 5 to 9 in your submission contain [map/satellite] images which may be copyrighted. [...]

The figures have been reworked. They are now based on the public domain data of "Natural Earth". In the "Data" section of the manuscript, we have added the following sentence: "In the plots, the coastline boundaries shown have been obtained from Natural Earth Version 4.2.0 (http://www.naturalearthdata.com/). "

Reviewer #1

In this manuscript. The authors just use a model, named as AWI-ESM, to discuss the extreme temperature change on global scale. In my opinion, the performace of global climate models in simulating the temperature changes show large uncertainties. Therefore, the uncertainty of the results in this manuscript could be large. The authors should use more outputs of global climate model to demonstrate the condifence of the results, and discuss the uncertainties of the results and methods. Due to this limitation, some minor comments and suggestions are not provided. The reviewer is looking forward to further improvement of this manuscript.

Thank you for your comment, we agree that it is better to use more than one data set. We have now used results of several global climate models and discussed similarities and differences of their results in the manuscript (see Section 4.2 and the Conclusions). The number of climate model data sets we could use is limited, however, since most model runs do not go until 2300. Besides that, the developed methodology is not restricted to a specific application, but it is also applicable to other data sets and other scientific questions regarding changes of extremes over time. Thus, the development of this methodology is a scientific contribution of its own right.

Moreover, many minor corrections have been made to the manuscript, and we have looked through the manuscript thoroughly to make additional improvements. We hope that with these changes, the minor issues you mentioned have also been addressed.

Reviewer #2

The study is focused on the statistical description of extreme monthly temperatures simulated by one global climate model spanning the period 1850 through 2300. The authors apply a non-inflationary generalized extreme value distribution and prescribe a sigmoidal temporal evolution of the distribution parameters. The rationale is that changes in these parameters will be slow from 200 onwards, increase the rate of change later and settle to a new equilibrium once the concentrations of atmospheric greenhouse gases are stabilized.
The main conclusion is that land areas experience in the simulation more drastic changes of the extreme values than ocean areas with the exception of the Arctic Ocean. This is probably due to the disappearance, of sea ice cover in this region. In general, a non-stationary GEV is able to describe the temporal evolution of the distribution of extreme monthly temperatures reasonably well.

Recommendation: In my opinion, the manuscript is generally well written, the approach is sensible and complements other studies on non-stationary extremes. There are, though, a few points that came drew my attention, and that the authors may want to consider in a revised version.

Main points
1) the study is focused on monthly mean temperatures. Although this a choice made by the authors, it is somewhat strange, as usually extreme temperatures are defined from daily means or weekly means (e.g. heat waves). The reader may be therefore disappointed that this is not the case. This needs to be made explicit in the title. Also, the rationale for this choice is not clear, as the daily data are surely available and would give a more interesting information.

We agree that daily data would be more suitable and we have now replaced the monthly data we originally used with several daily data sets that are analyzed and compared (following also a suggestion by the other rewiever). This has made it necessary to rewrite Sections 4.2 and 6 as well as parts of other sections. The results, however, are in general quite similar to those from the previous analysis, and although the predicted values are different now, most of the conclusions are still valid.

2) Although the text is generally well written, I would have wished sometimes a more accurate word choice. For instance, often the authors refer to models. The acquainted reader would be able to identify which types of models the authors are referring to (statistical, Earth System models..) but a more average reader would be slowed down when reading. I give some examples below.

Thank you for your comment, that wording could indeed have caused confusion. We have now made it more precise by adding "statistical" or "climate"/"earth system" model when necessary.

3) The quality of the figures in the reviewer's copy is rather low - this may be a problem of the rendering by the submission system, but starting in Figure 6 and especially Figure 10 are of rather low quality. Figure 10 is barely readable.

In the PDF, the figures are printed in low resolution by default by the submission system. For each figure in the file, there is a small link in the top-right corner, clicking on it will open the file in large resultion. In addition, we have now split up Fig 10 in four separate figures.

Particular points:

3) I would open a new paragraph in line 15

We have done that.

4) line 96: From the year 2100 on, CO2 emissions are assumed to be zero.

I guess that the climate simulation does not include a carbon cycle model, at least this is not mentioned. Thus, the concentrations of greenhouse gases are prescribed and set to a constant value, Figure 4 also suggests this. This is different from prescribing the emissions to zero.

You are right about that, and we have rewritten the text accordingly. It now reads: "For the years after 2100, the climate model runs with prescribed CO2 values that are set to the value of the year 2100."

5) At several instances the text refers to 'long term' and 'long timescales'. Please, be more specific on a long time-scale

We have replaced "long timescales" with "timescales spanning several centuries" in the abstract and elsewhere in the text.

6) Abstract and elsewhere 'Different models...' Please, specify (different statistical models)

We have now specified it in different parts of the texts, it should be more clear now.

7) ' In addition, our models differentiate between changes in mean, in variability and in distributional shape'

This can be a bit misleading, as the authors are referring to the mean, variability and shape of the GEV, but many readers will interpret this as changes in the mean, variability and shape of the original distribution of monthly temperatures. This is actually the most interesting point, whether for instance, changes in return values are caused by changes in mean temperature or by specifically changes in the tail of the distribution. In my interpretation this is not addressed in the study. Again., some readers may feel disappointed and this needs to be explicitly stated in the abstract.

You are right about that, that was a problem especially because that sentence was written in the abstract. We have changed the phrase into "In addition, our statistical models allow for changes in the different parameters of the fitted generalized extreme value distributions (a location, a scale and a shape parameter) to take place independently and at varying time periods."

8) line 14 ' Model simulations'. Please, specify Earth System models

This has been done as suggested.

9) 'Changes in the magnitude and the frequency of extreme events can be caused by changes in the mean values, in the variability, in the heavy-tailedness or by a combination of these factors'

We have replaced the sentence with "Geographically varying developments can be detected [...] with regard to [...] the extent to which the changes in extreme events are caused by changes in the location, the scale or the shape of the distribution of the annual maxima"

10) ' the distribution of the maximum of independent and identically distributed copies X1 , . . . , Xn of it'

Copies is not the right word, as their values are not identical

We have rewritten the sentence as: "Let X1,...,Xn be stochastically independent random variables, each having the same (unknown) probability distribution."

11) enumeration of equations ?

I think all equations should be numbered

All equations are now numbered (except for the equations describing the statistical models, because those models have their own designations).

12) line 385 : 2500 should read 2050 ?

It should indeed. As a result of the usage of a different data set in the revised version, the sentence has been changed completely. All year numbers in the revised version were double-checked to prevent similar typos.
